# Novel method to quantify peptidylarginine deiminase activity shows distinct citrullination patterns in rheumatoid and juvenile idiopathic arthritis

**DOI:** 10.3389/fimmu.2023.1111465

**Published:** 2023-01-30

**Authors:** Karen Yu, Luna Dillemans, Mieke Gouwy, Helena Bessa, Mieke Metzemaekers, Erik Martens, Patrick Matthys, Xavier Bossuyt, Patrick Verschueren, Carine Wouters, Lien De Somer, Paul Proost

**Affiliations:** ^1^ Laboratory of Molecular Immunology, Rega Institute for Medical Research, Department of Microbiology, Immunology and Transplantation, KU Leuven, Leuven, Belgium; ^2^ Laboratory of Immunobiology, Rega Institute for Medical Research, Department of Microbiology, Immunology and Transplantation, KU Leuven, Leuven, Belgium; ^3^ Laboratory Medicine, University Hospitals Leuven, Department of Microbiology, Immunology and Transplantation, KU Leuven, Leuven, Belgium; ^4^ Skeletal Biology and Engineering Research Center, Department of Development and Regeneration, KU Leuven, Leuven, Belgium

**Keywords:** citrullination, inflammation, joint inflammation, peptidylarginine deiminase, autoimmunity

## Abstract

**Introduction:**

Peptidylarginine deiminases (PADs) mediate citrullination, an irreversible posttranslational modification that converts arginine to citrulline residues in proteins. Rheumatoid arthritis (RA) is characterized by unique autoantibodies that recognize citrullinated peptides, which are highly specific for this disease. However, the mechanism preceding the anti-citrulline response remains largely unclear. PAD enzymes are known to fuel the autoimmune response by generating autoreactive epitopes, and sustain local synovial inflammation through neutrophil extracellular trap formation. Therefore, detecting endogenous PAD activity is important to understand the pathogenesis of arthritis.

**Methods:**

In this study, we improved a fluorescent in vitro assay to enable endogenous PAD activity characterization in complex samples. We combine the use of an in-house synthetic, arginine-rich substrate and a negatively charged dye molecule to visualize enzyme activity.

**Results:**

This pioneering PAD assay allowed profiling of active citrullination in leukocytes and in local and systemic samples of an arthritis cohort. Our results reveal that RA and juvenile idiopathic arthritis (JIA) synovial fluids display similar levels of PAD activity. In contrast, citrullination was limited in joints of patients suffering from gout or Lyme’s disease. Interestingly, in blood, a higher level of extracellular citrullination was only found in anti-CCP-positive RA patients.

**Discussion:**

Our finding suggests that enhanced synovial PAD activity drives the loss in tolerance towards citrullinated proteins and that systemic citrullination may indicate the risk for developing citrulline-specific autoimmunity.

## Introduction

Peptidylarginine deiminases (PAD) are calcium-dependent enzymes that convert arginine to citrulline residues in a posttranslational modification known as citrullination (1). In humans, five PAD isoforms, PAD1-4 and PAD6, have been identified and have isozyme-specific expression profiles in the human body. The ubiquitous expression of this enzyme family allows them to regulate many physiological processes including embryonal growth and cellular host defense, but also explains their marked implications in a broad spectrum of diseases (2–4). Citrullination is particularly relevant in rheumatoid arthritis (RA), where uncontrolled PAD activity gives rise to citrullinated antigens that become epitopes for anti-citrullinated protein antibodies (5). Additionally, PADs stimulate the release of neutrophil extracellular traps in the RA synovium, which is an alternative pathway that allows citrullinating enzymes to sustain local inflammation (6). These findings allude to an important role for PADs in RA pathology, though conclusive research on PAD enzymes as drivers of RA is scarce. Similarly, citrullination potentially plays a role in bacterial and viral sepsis as recent studies identified high levels of circulating citrullinated histone H3 in sepsis and corona virus patients (7, 8). However, the behavior and role of PAD enzymes upon infection remains elusive.

A significant complication in studying protein citrullination is the lack of detection techniques to measure PAD activity (9). To date, commercially available PAD assays suffer from limitations including compromised specificity, restricted dynamic sensitivity, incompatibility with detection in complex patient samples and high cost (10). In this article, we optimized a chemical probe for detection of PAD activity (11) in complex biological samples such as leukocyte lysates and clinical samples including serum, plasma and synovial fluid (11). We characterized extracellular PAD activity in synovial fluids and blood of adult and pediatric patients with inflammatory joint disorders. Our results detected distinct patterns of PAD activity within our cohort and provide unique evidence for its relation to the anti-citrulline humoral response in RA. Our data demonstrate the use of this PAD activity assay in clinical research and provide new insights into citrullination in inflammatory joint diseases.

## Materials and methods

### Chemical synthesis and purification of a fluorescent PAD substrate

Briefly, the fluorescently labelled peptide substrate was chemically synthesized on Rink amide resin using fluorenylmethoxycarbonyl (Fmoc) solid phase peptide synthesis (SPPS) on an Activotec P11 synthesizer (Activotec, Cambridge, U.K.). The peptide TAMRA-(GRGA)_4_ consists of four repeats of the arginine-containing tetrapeptide Gly-Arg-Gly-Ala and was N-terminally coupled to the fluorescent group 5(6)-carboxytetramethylrhodamine (TAMRA). After SPPS, the 2,2,4,6,7-pentamethyldihydrobenzofuran-5-sulfonyl (Pbf) side chain protection group on the arginines was chemically removed in a mixture containing 88.9% (v/v) trifluoroacetic acid (TFA, Biosolve, Valkenswaard, The Netherlands), 4.4% (v/v) thioanisole (Arcos Organics, Geel, Belgium), 2.2% (v/v) 1,2-ethanedithiol (Merck, Darmstadt, Germany), and 66.7 mg/mL crystalline phenol (Merck). The peptide was precipitated in 90% (v/v) cold methyl tert-butyl ether and purified through reversed-phase HPLC (Waters Corporation, Milford, MS), on a 250x8.0-mm PepMap C18 column (VDS Optilab, Berlin, Germany). Elution was performed in an increasing acetonitrile gradient in 0.1% (v/v) TFA. Peptides were detected and their relative molecular mass (Mr) confirmed *via* electrospray ion trap mass spectrometry (AmaZon SL, Bruker, Bremen, Germany). The purified peptides were lyophilized (Speedvac) overnight and stored at -20°C until use.

### Patient samples and healthy controls

Synovial fluids of patients with RA, juvenile idiopathic arthritis (JIA), gout or Lyme’s disease were collected in ethylenediaminetetraacetic acid (EDTA)-treated BD vacutainers (BD Biosciences, Franklin Lakes, NJ, U.S.A.). Each sample was centrifuged for 10 minutes at 400 g. Cell-free supernatant were collected and stored at -80°C. Platelet-free plasma was isolated after centrifugation of whole blood (collected in EDTA-treated vacutainers) for 20 minutes at 400 g, followed by 10 minutes of centrifuging the supernatant at 17000 g. Whole blood was collected in BD Vacutainers SST and centrifuged at 800 g for 10 minutes to isolate serum. Synovial fluids were only collected upon need for joint aspiration and were provided by the rheumatology unit of the university hospital UZ Leuven. Ethical approval was granted by the local Ethics Committee of the University Hospitals Leuven (UZ Leuven) under numbers ML1814, S61878 and S65508. Patients and healthy blood donors provided informed consent according to the ethical guidelines of the Declaration of Helsinki.

### Leukocyte isolation

For the isolation of peripheral blood mononuclear cells (PBMCs) and neutrophils, peripheral blood was taken from healthy volunteers and processed by density gradient centrifugation in a Pancoll (Pan-Biotech, Aidenbach, Germany) gradient. CD14^+^ and CD14^-^ cells were separated by positive selection with CD14 microbeads (Miltenyi Biotec, Bergisch Gladbach, Germany). Neutrophils were purified using the EasySep™ Direct Human Neutrophil Isolation Kit (Stemcell technologies, Vancouver, Canada). All isolation methods were performed according to the manufacturer’s instructions. Cells were counted in Türk’s solution using a Bürker chamber.

### Preparation of cell lysates and protein isolation

After isolation, cells were centrifuged for 5 min at 400 g and resuspended in RIPA buffer (Pierce, Thermofisher, Waltham, MA, U.S.A.) with 1% protease inhibitor cocktail (P8430, Sigma-Aldrich, St. Louis, MO, U.S.A.), following the ratio of 30 μL RIPA buffer per one million cells. Cells were shaken on ice for 15 minutes and subsequently centrifuged at 17000 g for ten minutes. Supernatant was collected and kept on ice or stored at -20°C until use.

### Dialysis of synovial fluids and protein measurement

To remove cell debris from samples, synovial fluids were first centrifuged for 5 minutes at 14000 g and subsequently desalted by gel filtration chromatography using Sephadex^®^ G-25 (Cytiva, Waltham, United States) columns in 15 mM Tris/HCl buffer (pH 7,4) or Zeba Desalting plates (Thermofisher) according to the manufacturer’s instructions. After dialysis, the absorbance at 280 nm was measured to quantify total protein content (BioTek, VT, U.S.A.).

### PAD activity assay

In all assays, in-house synthesized TAMRA-(GRGA)_4_ and Evans blue (UCB, Brussels, Belgium) were used as PAD substrate and fluorescence quencher, respectively. Various concentrations of substrate (0 μM – 80 μM) and quencher (0 μM – 120 μM) were included to determine their optimal range to monitor PAD activity in cell culture supernatant, plasma, serum and synovial fluid. Human recombinant PAD4 (Sf9) (0.03 nM-300 nM, Sigma-Aldrich) and rabbit skeletal muscle PAD2 (0.1 nM-100 nM, Sigma-Aldrich) were used to optimize this assay. Enzyme inhibitors used include 3 mM EDTA, EDTA-free protease inhibitor cocktail (P8340, Sigma-Aldrich), and the PAD inhibitor BB-Cl-amidine (20-30 µM, Cayman Chemicals, Ann Arbor, MI, U.S.A.). PAD assays were initiated by the addition of 3 mM CaCl_2_ and 1 mM dithiothreitol (DTT). PAD activity was monitored in black, clear flat bottom, 96-well plates (Greiner Bio One, Vilvoorde, Belgium) using a CLARIOstar plate reader (BMG Labtech, Ortenberg, Germany) at 555-580 nm. Enzyme velocity is expressed as relative fluorescence in function of time.

### Statistical and image analysis

Statistical analyses were performed and graphs were prepared using GraphPad Prism version 8 (GraphPad Software, San Diego, CA, U.S.A.). Normality of data was assessed with the Shapiro-Wilk test prior to further statistical analysis by unpaired t-test for assessing leukocyte subpopulations and one-way ANOVA analysis or Kruskal-Wallis analysis in patient cohorts. Linear regression was performed on raw data to obtain the slope representing enzyme activity over time and this is expressed by change in relative fluorescence units per minute (ΔRFU/min). Experiments were carried out in three experimental replicates for *in vitro* PAD activity assays. The boxplots are presented with median as center value, errors bars indicate min to max value. Significance was considered at p ≤ 0.05.

## Results

### TAMRA-(GRGA)_4_ is dose-dependently quenched by Evans blue

**Figure 1:**
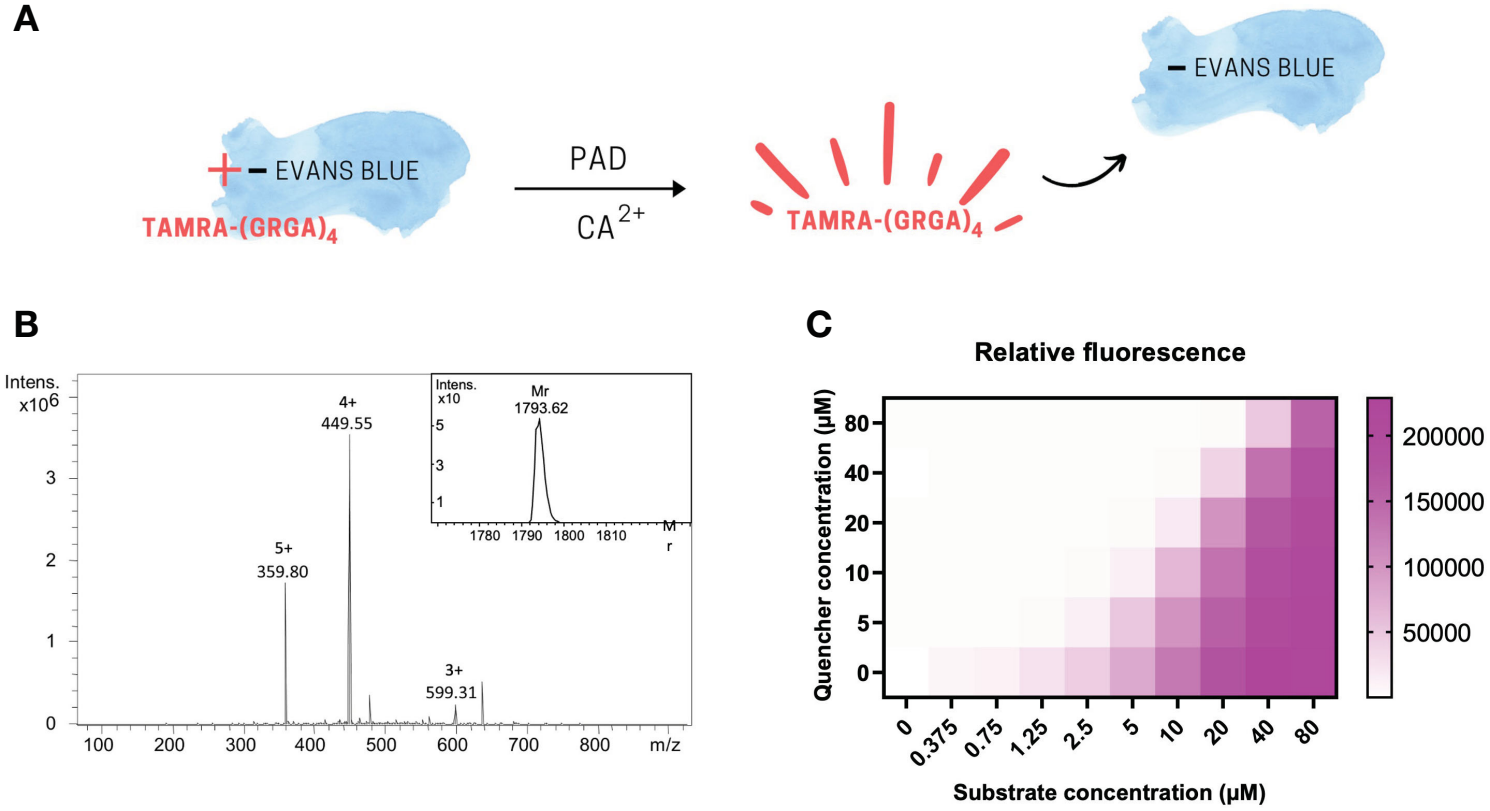
The PAD substrate TAMRA-(GRGA)_4_ is dose-dependently quenched by Evans Blue. Graphical scheme of fluorescence quenching principle **(A)**. After chemical synthesis of the fluorescent probe, the quality of the synthesized substrate TAMRA-(GRGA)_4_ was validated by ion trap mass spectrometry **(B)**. The experimentally determined relative molecular mass of 1793.62 was calculated from the multiple charged ions of the mass spectrum using Bruker deconvolution software and shown as insert on the unprocessed mass spectrum showing the multiple charged ions in panel **(B)** Quenching of TAMRA fluorescence was achieved in a dose-dependent manner by addition of Evans blue **(C)**.

The PAD substrate TAMRA-(GRGA)_4_ served as our fluorescent substrate (**Figure 1A**) and was obtained after chemical Fmoc peptide synthesis followed by HPLC purification, and its Mr was confirmed by ion trap mass spectrometry (**Figure 1B**). The experimentally determined Mr of TAMRA-(GRGA)_4_ was 1793.62 and corresponds to the theoretical average Mr value of 1793.20. The mass spectrum was devoid of contaminants, which indicated that the PAD substrate was of high purity and therefore suitable for further assay development. In a next step, we determined whether TAMRA-(GRGA)_4_ was quenched upon incubation with the negatively charged dye Evans blue (12). The results clearly show that Evans blue was able to induce a dose-dependent inhibition of fluorescence emitted by TAMRA-labelled substrate (**Figure 1C**).

### Method improvement to use TAMRA-(GRGA)_4_ as probe to detect active PAD in complex biological samples

**Figure 2:**
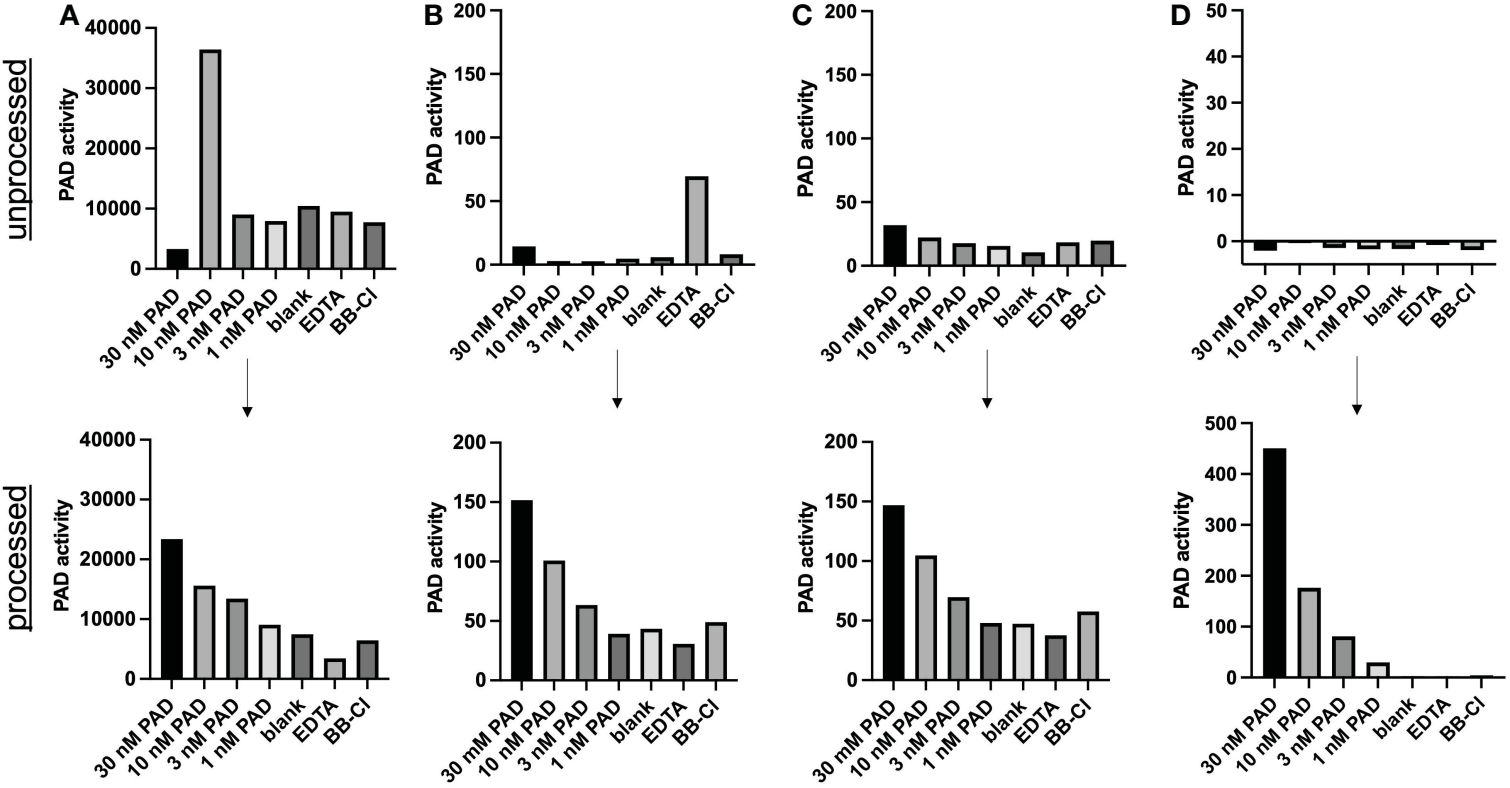
Method improvement for PAD activity detection in complex samples. PAD activity was detected through conversion of the TAMRA-(GRGA)_4_ substrate after addition of (30 nM-1 nM) recombinant PAD4 to heat-inactivated synovial fluid **(A)** plasma **(B)**, serum **(C)** or cell culture medium (RPMI) **(D)**. Unprocessed samples are shown in the top half of the figure (n=1). TAMRA-(GRGA)_4_ substrate conversion after desalting the samples (processed) through gel filtration chromatography is illustrated in the lower panels of the figure (one representative out of 3 or more experiments is shown). A quencher concentration adjusted according to protein content and a substrate concentration of 0.5 µM were used. PAD activity was inhibited by either 3 mM EDTA or 30 µM BB-Cl-amidine (BB-Cl). PAD activity is indicated as substrate conversion measured as change in relative fluorescence units per minute (ΔRFU/min).

TAMRA-labeled, arginine-containing peptides were suggested as chemical probes for detection of PAD activity (11). We spiked different doses of recombinant PAD in cell culture medium, serum, plasma and synovial fluid to assess this method for use in experimentally and clinically relevant samples (**Figure 2**). Spiking recombinant PAD in all samples generated fluorescent signals that were independent of the amount of active PAD (**Figures 2A–D**, top panels). We concluded that the method described as such failed to measure active citrullination in protein-rich samples. Nonetheless, we managed to overcome this complication by subjecting complex samples to gel filtration chromatography and by adjusting the quencher concentration to the total protein content of the sample of interest (**Figures 2A–D**, lower panels). To determine the optimal Evans blue concentration in future experiments, we made serial dilutions of EDTA-treated plasma as reference for complex samples and determined the minimal Evans blue concentration that quenches all fluorescence in the presence of 0.5 µM TAMRA-(GRGA)_4_ substrate. These quencher concentrations were plotted in relation to the absorbance at 280 nm as a measure of protein content, thus creating a standard curve (**Supplementary Figure 1**) (R2 = 0.96). This standard curve allows to determine the optimal quencher concentration for measurement of PAD activity in complex biological samples based on their total protein content.

### Natural rabbit PAD2 and recombinant human PAD4 are compatible with this PAD assay

**Figure 3:**
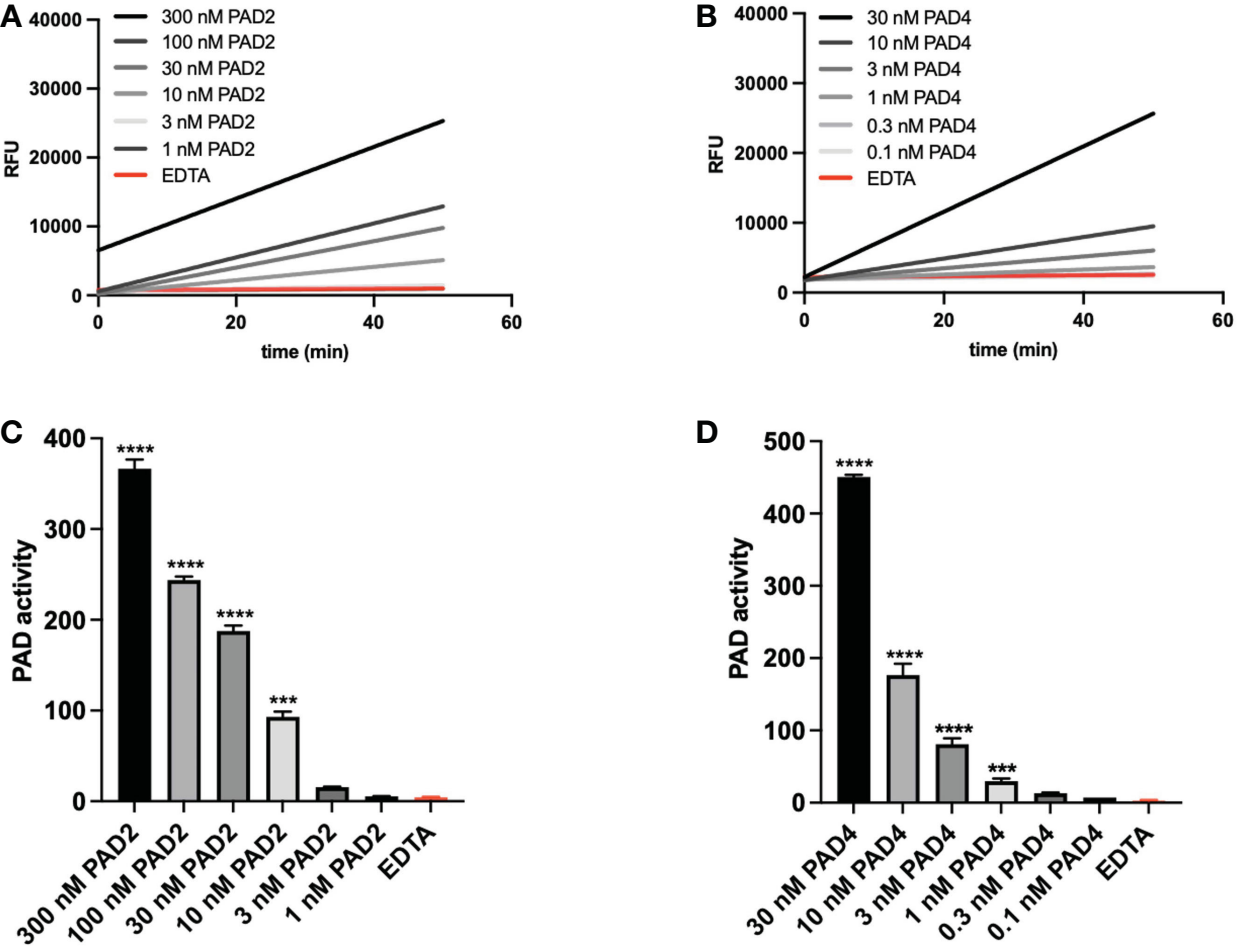
PAD activity can be measured sensitively and consistently. Natural rabbit PAD2 **(A–C)** and recombinant PAD4 **(B–D)** were used to determine assay characteristics such as variability and sensitivity. Real-time substrate conversion is illustrated in panels A and B (one representative experiment). Corresponding analyses of enzyme activity are depicted in panels **(C, D)** Enzyme activity (panels C and D) was calculated based on the slopes obtained after measuring the relative fluorescence for 50 minutes. A quencher concentration of 7.5 µM and a substrate concentration of 0.5 µM were used. Three mM EDTA was added as inhibitor of PAD activity. Enzyme activity refers to substrate conversion, detected as change in TAMRA fluorescence per minute (ΔRFU/min). Data in panels **(C, D)** are expressed as median change in fluorescence per minute ± standard error of the mean (ΔRFU/min). Significance considered *** for p<0.001 and **** for p< 0.0001 by one-way ANOVA.

Using the optimized method, we tested PAD2 and PAD4, the two most abundantly expressed isoforms of citrullinating enzymes (13), and determined the compatibility of this PAD activity assay with their detection. Different doses of natural PAD2 and recombinant PAD4 were tested in the activity assay and enzyme kinetics were shown by displaying PAD activity over time (**Figure 3**). We determined the enzyme activity based on the relative TAMRA fluorescence emitted after substrate conversion over time and before substrate saturation was reached. Both enzyme isoforms were quantifiable in a dynamic range of at least 30 and suffered from very little variability between experimental repeats (n=3) (**Figures 3C, D**).

### Higher PAD activity in myeloid compared to lymphoid cells

**Figure 4:**
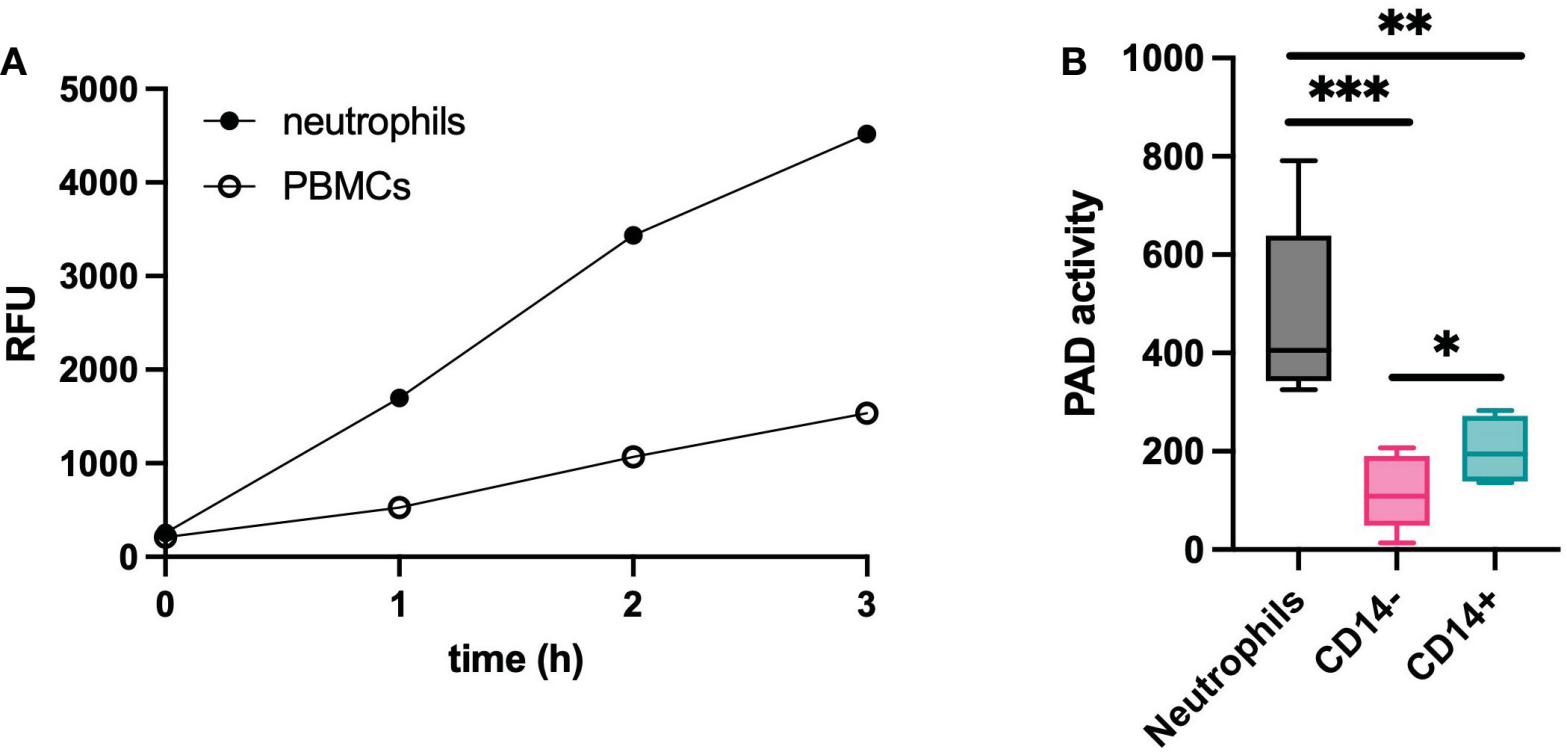
PAD activity in leukocytes. Lysates of leukocytes (1x10^6^ cells) from healthy subjects (n=6) were used to determine the citrullinating capacity per cell type. Substrate conversion by PAD from neutrophils and PBMCs is expressed in relative fluorescent units (RFU) and was monitored over time **(A)**. Further characterization was performed on neutrophils and PBMC-derived cells that were selected based on presence or absence of CD14 expression **(B)**. An Evans blue quencher concentration of 10.5 µM and a TAMRA-(GRGA)_4_ substrate concentration of 0.5 µM were used. PAD activity in indicated as substrate conversion detected as change in relative fluorescence units per minute (ΔRFU/min). Error bars show min to max value, center values represent median value. Significance considered * for p<0.05, ** for p<0.01, *** for p<0.001 by unpaired t-test.

According to recent ‘omics’ data, leukocytes are expected to express significant levels of PAD (14). Therefore, we isolated fresh neutrophils and PBMCs from healthy donors to characterize their enzyme activity. In **Figure 4A**, active citrullination of the TAMRA-(GRGA)_4_ substrate is depicted over a time course of three hours for both cell populations. Our data showed that neutrophil-derived cell lysates contained higher levels of PAD activity compared to PBMCs. To characterize the PAD activity in different mononuclear cells, PBMCs were further purified based on CD14 expression. CD14 positive monocytes expressed more active PAD than CD14 negative cells, but contained significantly less PAD compared to neutrophils (**Figure 4B**). According to these results, the amount of potentially available PAD activity is the highest in neutrophils and the activity observed in PBMCs is mostly attributable to CD14 positive monocytes.

### Extracellular citrullination detected in synovial fluids and plasma of patients with arthritis

**Figure 5:**
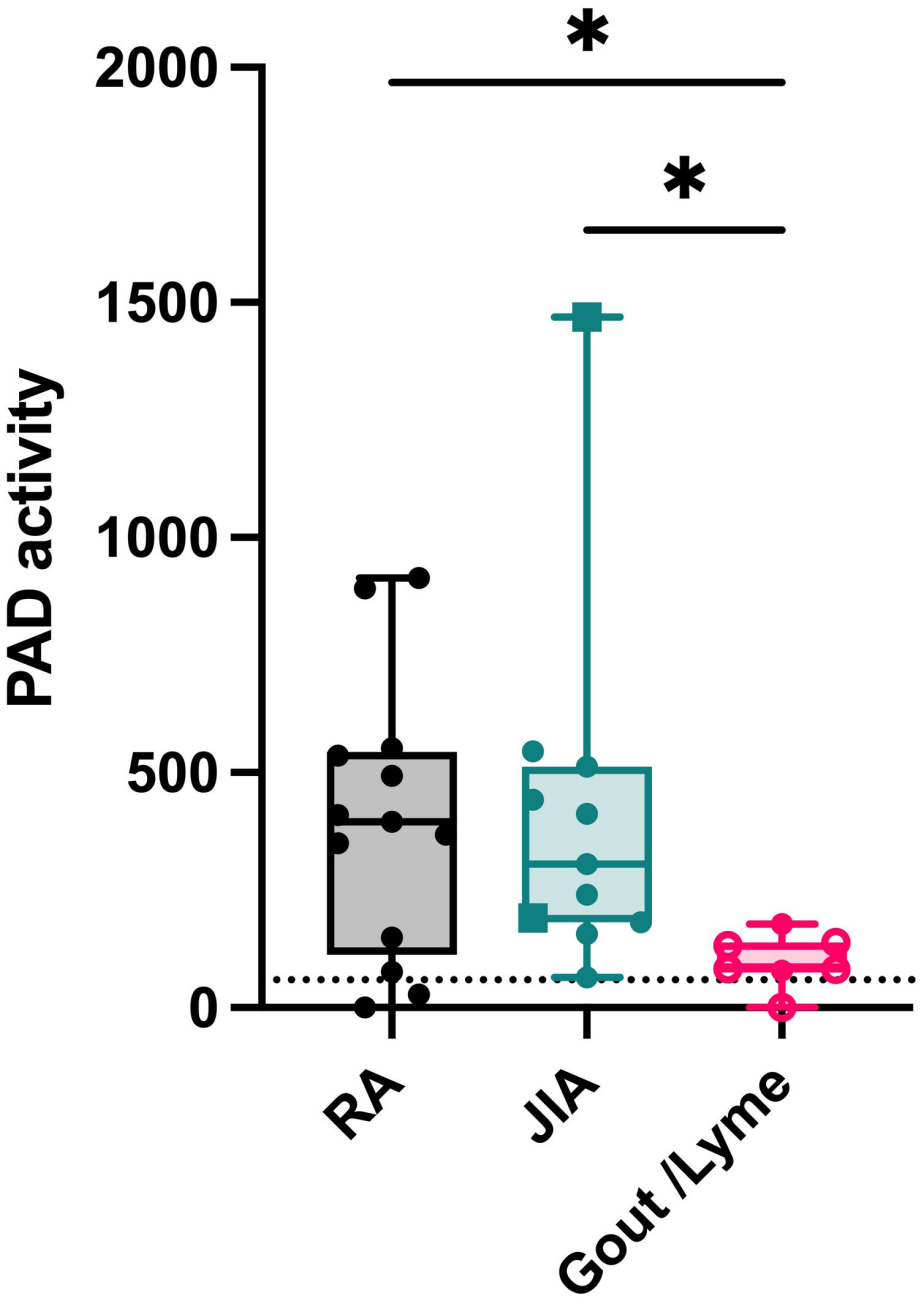
Extracellular citrullination in synovial fluids of patients with RA and JIA. PAD activity was detected in synovial fluids from patients diagnosed with rheumatoid arthritis (RA, n=12), Juvenile Idiopathic Arthritis (circles, oligoarticular JIA, n=8; rectangles, polyarticular JIA, n=2) or gout (open circles, n=4) and Lyme’s Disease (n=2). An Evans blue quencher concentration of 75 µM and a TAMRA-(GRGA)_4_ substrate concentration of 0.5 µM were used. Error bars show min to max value, center values represent median value. Significance considered * for p<0.05 by Kruskal-Wallis. The detection limit in synovial fluid was determined by inhibition of PAD activity through EDTA treatment (59 ΔRFU/min, dotted line).

**Figure 6:**
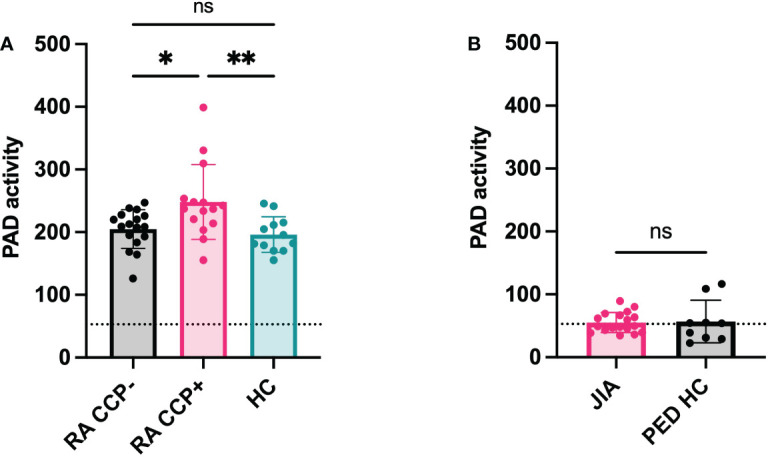
Systemic citrullination in anti-cyclic citrulline antibody positive RA patients. PAD activity was detected in sera of anti-cyclic citrulline peptide antibody (CCP) positive Rheumatoid Arthritis (RA) patients (n=15) and anti-CCP negative RA patients (n=17) and compared to healthy subjects (n=12) **(A)**. In parallel, PAD activity was measured in plasma of Juvenile Idiopathic Arthritis (JIA) patients (n=18) and compared with plasma of healthy pediatric controls (n=9) **(B)**. An Evans blue quencher concentration of 55 µM and 75 μM were used for serum and plasma detection, respectively, in combination with a TAMRA-(GRGA)_4_ substrate concentration of 0.5 µM. Error bars show min to max value, center values represent median value. Significance considered * for p<0.05, ** for p<0.01 by one-way ANOVA Tukey’s multiple comparisons test for RA samples and Mann-Whitney test for JIA samples. Detection limit in sera determined by EDTA treatment and represented by dotted line (71 ΔRFU/min for sera, 53 ΔRFU/min for plasma). ns, not significant.

RA, JIA, gout and Lyme’s disease are all characterized by joint inflammation and share similarities such as a high neutrophil infiltration and the release of proinflammatory cytokines in the joints. Still, citrullination seems distinctly relevant in RA pathology due to the presence of anti-citrullinated peptide antibodies (15). However, it remains uncertain whether the level of PAD activity in the joints or in circulation could be a contributing factor to the disease. Therefore, we measured active citrullination in inflamed joints of a patient cohort that comprises different arthropathies. RA and JIA patients expressed high levels of citrullination in synovial fluid whereas PAD activity was nearly absent in synovial fluids of gout and Lyme’s disease patients (**Figure 5**). Surprisingly, JIA synovial fluid samples displayed similar levels of PAD activity compared to fluids in joints of patients with RA. This finding provides proof for extracellular citrullination in synovial fluids of patients with JIA in addition to RA. Moreover, these data suggest that RA, but also JIA, involve a citrullination pathway that remains inactive in gout and Lyme’s disease, two pathologies which are also characterized by a high influx of neutrophils in inflamed joints (16, 17). As we observed local extracellular citrullination in the joints of RA and JIA patients, we continued this study by characterizing systemic citrullinating activity in these patients (**Figure 6**). In addition, we stratified patients with RA based on CCP serology to clarify the relevance of PAD activity in sera for the autoimmune response. Our data showed enhanced PAD activity in the sera of RA patients with circulating anti-CCP antibodies (**Figure 6A**), while a comparable amount of PAD is present in anti-CCP negative RA patients and healthy controls. Moreover, plasma samples from patients with JIA displayed low levels of PAD activity, which were similar to plasma values in healthy pediatric controls (**Figure 6B**).

## Discussion

We optimized a fluorescent citrullination method to a functional PAD activity assay that is compatible with complex biological samples. Firstly, we improved assay performance by eliminating contaminating ions such as salts from samples with gel filtration chromatography. Secondly, we realized that for the assay to work with protein-rich samples a higher concentration of Evans blue was required to compete with larger interfering molecules such as albumins (12). With the addition of these crucial steps (sample desalting and quencher concentration adjustment), we obtained dose-response curves in all biological settings, thus verifying the compatibility of the optimized PAD activity assay with biological samples.

Using this novel method, PAD activity was detected in leukocytes, where a higher level of activity in neutrophils was found compared to PBMCs. We further investigated this difference by separating the PBMCs by CD14 selection and concluded that lymphocytes, which account for 90% of PBMCs, contained only a limited citrullinating capacity. In contrast, CD14 positive monocytes showed higher levels of PAD activity, though still lower compared to neutrophils. The difference in activity could reflect the lack of PAD4 protein expression in monocytes (18). In contrast to monocytes, neutrophils express both PAD2 and PAD4 at the protein level which may explain the faster rate of substrate conversion (19). For the first time, we characterized the potential citrullination capacity of the most important leukocyte subtypes in healthy subjects. Neutrophils are important mediators of citrullination, especially during acute inflammation where they serve in our first line of defense. In fact, NETosis, an essential microbial killing mechanism of neutrophils, can be triggered by PAD (20, 21). Citrullinated histone H3 for example, is widely used as a marker for NET release and often associated with a higher degree of inflammation. We believe that this PAD assay could serve as a helpful tool in elucidating the biological interplay between PAD, NETosis and the elicited inflammatory effects.

In our cohort of arthritis patients, we found distinct patterns of endogenous PAD activity in the joints and blood of these patients. Our data showed that synovial fluids from patients with RA and JIA contain more citrullinating capacity compared to those of gout and Lyme’s disease patients. Remarkably, the joints of JIA patients displayed similar levels of enzyme activity compared to RA samples. It provides, for the first time, evidence for extracellular citrullination in synovial fluids of JIA patients. Furthermore, the lack of active PAD enzymes in gout and Lyme’s disease samples suggests that citrullination is not related to joint inflammation in general or infiltration of neutrophils in particular. Instead, we suspect that increased levels of synovial PAD activity result in local saturation of citrullinated proteins, which in turn may fuel an anti-citrulline response. This is also supported by our finding that anti-CCP positive RA patients were characterized by higher levels of PAD activity in serum. Although JIA patients did not display high plasma PAD activity, these patients may reside in a pre-humoral stage of the disease where local PAD activity has not broken the tolerance towards citrullinated proteins. Excessive citrullination probably precedes the development of autoantibodies, therefore it is plausible that the serology at this stage of the disease is inefficient to determine (future) reactivity towards citrullinated proteins (22, 23). The fact that some patients with polyarticular JIA are prone to develop a typical RA phenotype later in life supports this hypothesis (24). Therefore, we believe elevated PAD activity may represent the risk of citrulline intolerance and serve as a novel biomarker for this subset of JIA patients, as early detection of anti-CCP can improve treatment (22).

In conclusion, we optimized and validated a functional PAD assay for use in patient samples. With this PAD assay, we gained insight into arthritic disorders by measuring active citrullination at the cellular level, locally at the site of inflammation and systemically in plasma and sera. We believe this novel PAD assay holds great potential and encourage further exploration of this assay in clinical and translational research.

## Data availability statement

The raw data supporting the conclusions of this article will be made available by the authors, without undue reservation.

## Ethics statement

The studies involving human participants were reviewed and approved by the Ethics Committee of the University Hospitals Leuven. Written informed consent to participate in this study was provided by the participants’ legal guardian/next of kin.

## Author contributions

KY wrote the first draft of the manuscript; KY, LD, MG, HB, MM, and EM performed experiments; MG, XB, PV, CW, and LS collected clinical samples; KY and PP designed the experiments and all authors critically reviewed and corrected the manuscript. All authors contributed to the article and approved the submitted version.
