# Supplementary figures and images for: Novel method to quantify peptidylarginine deiminase activity shows distinct citrullination patterns in rheumatoid and juvenile idiopathic arthritis
Source: Front Immunol. 2023 Jan 30;14:1111465. doi: 10.3389/fimmu.2023.1111465 (PMC9923157; doi:10.3389/fimmu.2023.1111465)

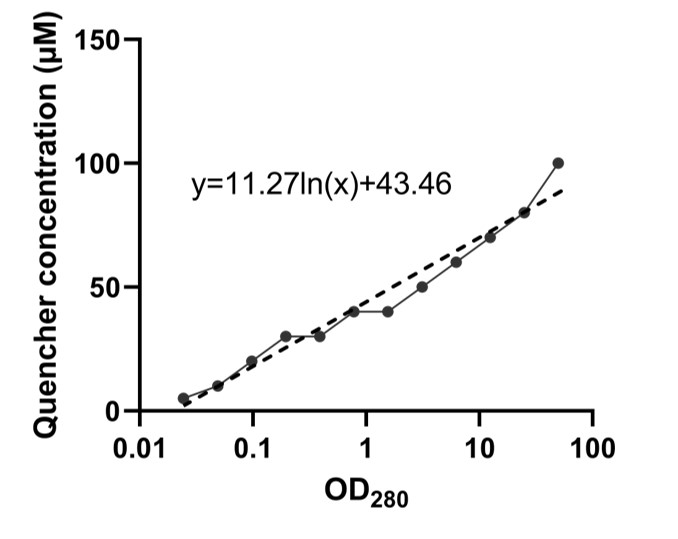

Supplement: Supplementary file 1 [file Image_1.jpeg]
