# Supplementary material for: Novel method to quantify peptidylarginine deiminase activity shows distinct citrullination patterns in rheumatoid and juvenile idiopathic arthritis
Source: Front Immunol. 2023 Jan 30;14:1111465. doi: 10.3389/fimmu.2023.1111465 (PMC9923157; doi:10.3389/fimmu.2023.1111465)
Supplement: Supplementary file 2 [file DataSheet_1.pdf]

Supplementary Table 1. Characteristics of patients with juvenile idiopathic arthritis.

Patient number	Patient identifier	diagnosis	age (years)	treatment at moment of sampling
**1**	MM020	oligoarticular JIA (ANA-)	14	NSAIDs
**2**	MM028	oligoarticular JIA (ANA+)	11	NSAIDs
**3**	MM032	oligoarticular JIA (ANA-)	8	NSAIDs
**4**	MM033	oligoarticular JIA (ANA+)	11	MTX 15 mg/w, NSAIDs
**5**	MM034	polyarticular JIA (ANA+)	12	none
**6**	MM041	oligoarticular JIA (ANA-)	4	NSAIDs
**7**	MM049	oligoarticular JIA (extended; ANA+, RF-)	14	NSAIDs
**8**	MM050	polyarticular JIA (ANA-)	11	MTX, NSAIDs, medrol 2 mg
**9**	MM052	polyarticular JIA (ANA+)	10	none
**10**	MM053	oligoarticular JIA (extended; ANA+)	16	none
**11**	MM058	oligoarticular JIA (ANA+)	16	NSAIDs
**12**	MM059	oligoarticular JIA (ANA-)	12	NSAIDs, Rilatine
**13**	MM060	polyarticular JIA (ANA+)	13	none
**14**	MM061	oligoarticular JIA (ANA+)	8	methotrexaat 15 mg/w, medrol 0,1 mg/kg/d
**15**	MM063	oligoartricular JIA (ANA+)	5	MTX SC
**16**	MM065	oligoarticular JIA (extended; ANA+, RF-)	15	Orencia and MTX SC
**17**	MM066	polyarticulaire JIA (ANA-, RF- with uveitis and cutaneous mastocytosis)	17	Humira, MTX and montelukast
**18**	MM072	unknown	unknown	unknown

Abbreviations: ANA, anti-nuclear antibodies; JIA; juvenile idiopathic arthritis; MTX, methotrexate; NSAID, non-steroidal anti-inflammatory drugs; RF, rheumatoid factor;
